# Dental image enhancement network for early diagnosis of oral dental disease

**DOI:** 10.1038/s41598-023-30548-5

**Published:** 2023-03-31

**Authors:** Rizwan Khan, Saeed Akbar, Ali Khan, Muhammad Marwan, Zahid Hussain Qaisar, Atif Mehmood, Farah Shahid, Khushboo Munir, Zhonglong Zheng

**Affiliations:** 1 Department of Computer Science and Mathematics, Zhejiang Normal University, Jinhua, 321004 Zhejiang China; 2 School of Computer Science, Huazhong University of Science and Technology, Wuhan, China; 3 Department of Computer Science, National University of Modern Language, NUML, Islamabad, Pakistan; 4 Division of Biomedical Imaging, Department of Biomedical Engineering and Health Systems, KTH Royal Institute of Technology, Stockholm, Sweden; 5 Department of Computer Science, University of Agriculture, Sub-Campus (Burewala-Vehari), Faisalabad, Punjab Pakistan; 6 Department of Radiology and Diagnostic Imaging, University of Alberta, Edmonton, Alberta Canada; 7 Key Laboratory of Intelligent Education Technology and Application of Zhejiang Province, Zhejiang Normal University, Jinhua, 321004 Zhejiang China

**Keywords:** Dentistry, Oral diseases, Dental diseases, Dental caries

## Abstract

Intelligent robotics and expert system applications in dentistry suffer from identification and detection problems due to the non-uniform brightness and low contrast in the captured images. Moreover, during the diagnostic process, exposure of sensitive facial parts to ionizing radiations (e.g., X-Rays) has several disadvantages and provides a limited angle for the view of vision. Capturing high-quality medical images with advanced digital devices is challenging, and processing these images distorts the contrast and visual quality. It curtails the performance of potential intelligent and expert systems and disincentives the early diagnosis of oral and dental diseases. The traditional enhancement methods are designed for specific conditions, and network-based methods rely on large-scale datasets with limited adaptability towards varying conditions. This paper proposed a novel and adaptive dental image enhancement strategy based on a small dataset and proposed a paired branch Denticle-Edification network (Ded-Net). The input dental images are decomposed into reflection and illumination in a multilayer Denticle network (De-Net). The subsequent enhancement operations are performed to remove the hidden degradation of reflection and illumination. The adaptive illumination consistency is maintained through the Edification network (Ed-Net). The network is regularized following the decomposition congruity of the input data and provides user-specific freedom of adaptability towards desired contrast levels. The experimental results demonstrate that the proposed method improves visibility and contrast and preserves the edges and boundaries of the low-contrast input images. It proves that the proposed method is suitable for intelligent and expert system applications for future dental imaging.

## Introduction

Dental image analysis is an important tool for detecting and diagnosing oral and dental diseases. Infectious microbiological diseases (e.g., dental caries, dental plaque, etc.) result in the parochial disintegration and annihilation of the compact ossified tissues. The most common causes of these dental diseases are associated with lifestyle factors and appear irrespective of age, caste, creed, sex, and location. In general practice, intraoral X-rays are often utilized when the patients are exposed to ionizing radiation^1^. It provides a limited angle for the view of vision. Effective dental lesion detection technologies can determine the incipient carious lesion with the help of effective changes in the tooth surface^2^. In most of the common dental diseases, an early-stage detection provides a better assessment for the diagnosis^3^ and also limits the overall cost and complications^4^. Moreover, the improvement in the visual quality of the captured images can assist significantly in improving associated tasks such as segmentation^5^, computer-assisted oral and maxillofacial surgeries^6^ and many image-guided robotics and intelligent expert system application^7^ tasks.

Images captured with digital devices for preliminary diagnosis encounter low contrast and defy camera settings to handle the issues and give rise to new challenges. In the case of dental spectral imaging, optical devices capture the images by using a light ring and a mobile spectral camera. Spectral imaging can record the reflection spectrum of the sample by using a ring illuminator. However, it limits the illumination intensity and reflection reaching the camera^8^. It also raises the acquisition time and demands hardware base adjustments (e.g., polarizers), resulting in over-exposure and under-exposures.

The input images captured with the digital devices require an adaptive enhancement operation to assist the early diagnosis besides the surgical operations in several dental and oral diseases^1^^9^. The extant contrast enhancement methods for medical images are based on histogram equalization^10^. The classic solutions^11^, and deep learning-based solutions^12^^13^ in medical imaging, and computer vision^14^ rely on image pairs or large scale datasets and provide a fixed balance of contrast with limited adaptability towards the nascent conditions. Moreover, learning a new task from the beginning relies on a tedious training process, raising the computational complexity and overall cost in practical scenarios. Thus, new adaptive solutions are required to resolve these challenging problems to improve the performance of practical medical applications. The quality of the captured images is degraded due to several factors, including acquisition techniques and limitation of the processing techniques^15^. Contrast enhancement techniques, such as histogram equalization (HE), contrast limited adaptive HE^10^^16^ and several follow up of these methods^17^ are widely used for the pre-processing of the medical images. Feature enhancement based on illumination weighting (FEW)^18^, simultaneous reflection and illumination enhancement (SRIE)^19^, and robust retinex-based simultaneous reflection and illumination (SLIMER)^20^ methods are proposed in the literature but fail in the case of robust changes.

The recent decomposition-based approaches^21^, deep retinex^22^, kindling the darkness method (KinD)^23^ and beyond brightening the low light images (BBLLI) method^14^ are fundamentally dependent on a large scale dataset of images pairs. The dependency of these methods on a large scale, carefully designed, and even paired training images dataset limits these methods’ practical implication in the target domain. The improvisation in these techniques may significantly contribute to improving the performance of many deep learning-based methods^12^^5^ including the improvement in the performance of the visual tracking and surgical robots^24^. Moreover, in the target domain, the enhancement techniques help the physician for better assessment and early diagnosis, which saves the additional complications and overall cost^17^. It is important to note that the two-dimensional representation of the three-dimensional object superposed the information. The challenges are more comprehensive than before when it comes to enhancing medical images. More adaptive and freestyle enhancement techniques are imperative to improve the performance of the associated tasks. Therefore, our work aims to provide an adjustable tool for enlightening dark images instead of providing fixed results.

In order to address the above-mentioned issues, we propose a novel clinically oriented contrast enhancement strategy to improve the visual quality of low-quality medical images. To the best of our knowledge, it is the first approach in the target domain. The proposed approach provides user-specific freedom of choice for luminance and contrast enhancement. A denticle edification network, denoted as Ded-Net, is end-to-end trained to obtain high-quality output images. The process is initialized by separating the reflection and illumination of the input images. In general, the direct image enhancement methods^25^^26^ amplify the artifacts in some low-lighting scenarios, which get amplified at every step during enhancement and might result in vulnerable image quality. In contrast, we propose independent enhancement operations to remove the reflection irregularities and inconsistencies of illumination distinctively. In our method, the total variation operation is optimized following the proposed clinically oriented enhancement strategy to preserve the input images’ strong edges, boundaries, and structure. The main contributions of the proposed work can be summarized as follow:

- In this paper, we present a novel contrast enhancement technique to improve the visual quality of dental images obtained from the spectral images dataset. Our method handles the challenges of ill-posed image decomposition and preserves the strong boundaries and edges without amplifying the hidden degradations in the low-quality input images and producing high-quality output images.
- A deep learning-based framework denoted as Ded-Net is proposed to improve the visual quality of the low-exposure input images. In our method, we pre-processed the spectral images and utilized only a few dental spectral images (i.e., 216 images only) for training our Ded-Net, and optimize the total variation loss function following image decomposition.
- Extensive experimental results based on subjective and objective evaluations demonstrate that our method outperformed the extant techniques. The proposed method improves the contrast and quality of poor-quality dental images and can also boost the performance of detection and segmentation besides early diagnosis of many dental diseases.

**Figure 1:**
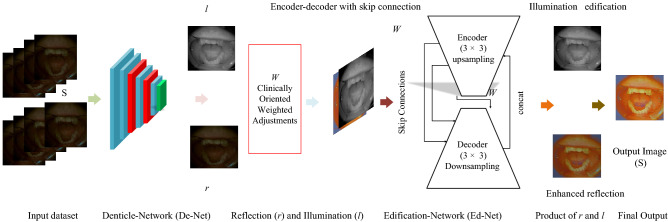
Framework of the proposed Denticle Edification network (Ded-Net).

## Related work

High-quality images are an essential part of modern intelligent applications in the domain of computer vision and image processing. But the images captured in robust environmental conditions often suffer from several artifacts due to the limitation in the capturing devices, camera settings, or variations in lighting conditions. Several methods have been proposed in the literature to encounter these artifacts and improve the images’ visual quality. These methods can be described mainly as direct enhancement methods^27^, retinex based methods^22^, deep learning-based methods^28^, and dehazing methods^29^. The retinex-based^30^ methods received comparatively more attention and have several applications^31^. These methods consider dividing the input image into reflection and illumination components to independently handle the associated artifacts^32^^23^. Given different illumination qualities, the ideal assumption that treats the reflection component as the enhanced result does not always apply, which could result in unrealistic enhancement like loss of details and distorted colors^33^.

The recent deep learning-based solutions outperform traditional approaches in terms of accuracy, robustness, and speed and gaining more attention. The deep learning-based methods can be classified as supervised learning-based methods, which utilize the image pairs for the training process^22^^4^, and unsupervised learning-based methods, which work without paired training dataset^34^^35^. Moreover, many other methods, including semi-supervised learning^36^ and deep reinforcement learning^37^ based methods, have also been proposed in the literature. Some methods consider the illumination information and proposed illumination-based weighted feature fusion methods^18^. Considering the illumination inconsistencies, decomposition-based methods are proposed for independent processing^32^ based on retinex theory^38^. Following the retinex theory, a lowlight image enhancement method LIME^39^ is proposed with illumination smoothness and prior information. A robust retinex-based SLIMER^20^ method is proposed to improve enhancement while encountering minor noises.

The worst form of noise in the lowlight regions distorts the global structure of the images. A joint enhancement and denoising (JED)^40^ method was proposed to counter noises. But the, denoising before enhancement produces blurry results, whereas after enhancement, it removes some of the important features. The learning-based restoration method (LBR) for the back-lit images^41^ is proposed to independently use front and backlit regions with an SVM classifier. On each of these regions, the optimal contrast tone mapping^42^, the mechanism can act to expose the under-exposed regions. Similarly, multiview cameras system^43^ based methods have also proposed to enhance the contrast^44^ by using a single image. However, its a complex process and has a higher computational complexity. The deep learning-based methods, deep-retinex^22^, kindling the darkness (KinD)^23^, beyond brightening lowlight images (BBLLI), deep dark to bright view (D2BV-Net)^32^ divide to glitter strategy^45^, retinex-inspired unrolling with cooperative prior architecture search for low-light image enhancement (RUAS)^36^, Zero-DCE^46^, attention guided lowlight image enhancement (AGLLIE)^47^ and Enlighten-GAN^34^ have been proposed recently to encounter the aforementioned enhancement challenges.

The existing learning-based methods demand a large-scale and paired training dataset, whereas some others require a careful selection of the images, which raises the overall cost and latency of the network. In our method, we handle these challenges and produce significant results without large-scale and careful selection of data.

## The proposed method

In this work, we propose an effective and practical method to improve the visual quality of the images captured with digital imaging devices, to compensate against the degradations of the poor quality images. The framework of our method is shown in Fig. 1. It demonstrates that a De-Net, based on several layers to separate the illumination (*l*) component from the reflection (*r*) component for input image (*S*), and subsequent enhancement operations are performed to compensate against the glitches of ill-posed image decomposition.

1 $$\begin{aligned} S= r\cdot l \end{aligned}$$

In order to avoid the amplification of the undesired artifacts (noise, texture, and structure), successive enhancement operations are embedded in the network to adjust the irregularities and inconsistencies of *r* and *l* components, respectively. Adaptive illumination enhancement operations are performed in encoder-decoder-based Ed-Net. In our method, we optimize the total variation operation to mitigate the structure and texture distortions and preserve the strong boundaries and edges. Finally, an inverse operation produces the visually pleasing output image. The proposed Ded-Net provides user-specific freedom of luminance in the final image without passing through the tedious training task.

**Figure 2:**
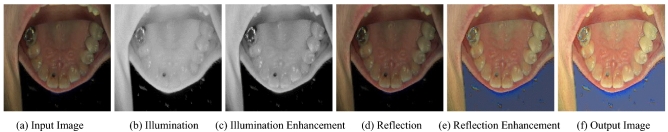
The decomposition of the input into reflection and illumination with respective enhancement operations.

### The proposed clinically oriented enhancement operations on reflection and illumination

In this work, we use the oral and dental spectral image database (ODSI-DB) dataset^8^, where it is a challenging task to capture such images without illumination and reflection frailties. The poor quality of the captured images often leads to several complications and hinders the early diagnosis of dental diseases. We proposed decomposing the captured images into reflection and illumination components to remove the irregularities of the reflection and illumination. We adjust the decomposition with a clinically oriented enhancement strategy and remove the undesired artifacts by embedding the proposed strategy in the deep network. The input images *S* of the subject, captured with radiance *r*, global light component *a* and light transmission component *l*, depict the image formation^29^ with *x*, *y* pixel coordinates to formulate proposed scheme as follow:

2 $$\begin{aligned} {S(x,y)}={r(x,y)} \cdot {l(x,y)}+{a}\ (1\hbox {-}{l(x,y)}) \end{aligned}$$

Moreover, capturing an image gives rise to several irregularities due to many uncontrollable factors, including lighting conditions and device capacity. These inefficiencies give rise to structure and texture irregularities when it comes to handling reflection and illumination. Where ‘*a*’ is measured with an estimation of $0.1 \%$ darkest pixels in the dark channel of *S*. For the reflection component, the intensity of brightness in a patch $\omega (x)$, centered at *x*, *y* is defined below for R, G, and B channels, with a patch size *p* of ${3 \times 3}$.

3 $$\begin{aligned} r^{dark}(x, y)= \min _{c\in \{r,g,b\}}r^{c}(x, y) \end{aligned}$$

In the target problem, the input images are captured in a scenario where a ring illuminator and the camera are mounted on a platform that slides over the main platform. The subject’s movement is reduced during imaging in combination with the main optical imaging platform. For an 8-bit haze image, whiteness depicts most of the pixels have maximum intensity (i.e., maximum N = 255), which is why it appears to be white. Thus, the darkening benefits the haze image. Consequently, the residual image is estimated as *N* - *S*(*x*, *y*) for low light and haze images. It shows that images in such conditions share the *l*(*x*, *y*) for 8-bit images. Thus the darkest regions contrast is possible to extract with an effective weighting strategy. The inversion of the decomposition components produces a residue image following the proposed weighting strategy for reflection ${{\dot{r}}{(x,y)}=1-r(x,y)}$, and illumination ${{\dot{l}}{(x,y)}=1-l(x,y)}$ components. The images captured from different viewing angles contain underexposed areas with low-intensity pixels in the backlighting regions. In such cases transmission coefficient with the lighting percentage reaching the camera is determined for optimal contrast adjustment.

4 $$\begin{aligned} l(x,y) = 1-\min _{p\in \omega (x,y)} \left( N- \max _{c}\frac{ N-S^{c}(x)}{a^{c}} \right) , c\in \{r, g, b\} \end{aligned}$$

Where the channel, ${c\in \{r, g, b\}}$. In order to provide the effective balance of luminance in the target scenario, we adjust the reflection and illumination components distinctively with the help of a clinically oriented contrast enhancement strategy as shown in Fig. 2. The reflection regularization weighting is proposed to mitigate reflection irregularities, and illumination weighting is proposed to maintain the per-pixel illumination consistency.

**Figure 3:**
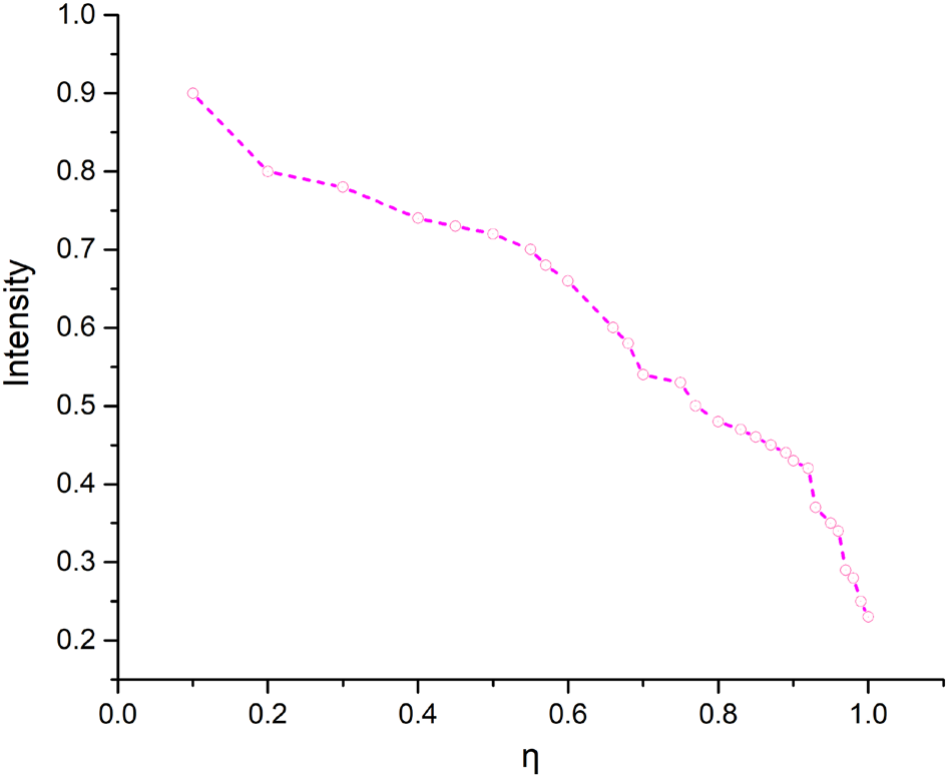
The effects of proposed enhancement parameter ${\eta }$ on intensity.

**Figure 4:**
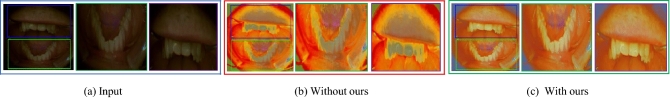
(**a**) Input images are shown with zoomed fragments, (**b**) shows the results without-ours method and (**c**) shows the results with ours method.

The proposed weighting parameters for the illumination ${w_l}$ and reflection ${w_r}$ provide strict control over the reflection and illumination. The respective adaptive adjustments reveal the hidden details of the underexposed pixels to revamp the balance of luminance.

5 $$\begin{aligned} \begin{aligned} w_l= \frac{exp^{l(x,y)}}{n} \\ w_r= \frac{exp^{\lambda -r_{(x,y)}\over {m}}}{\eta } \end{aligned} \end{aligned}$$

Where, ${0< n, \eta \le 1}$, and ${0< \lambda \le 255 }$ and ${0< m \le 255}$, provide spatial adjustments. In Eq. (5), the values of *n* and ${\eta }$ provide control over the brightness. We conduct extensive experiments to demonstrate the effectiveness of these parameters and find out that middle values are much more suitable for optimal results. Moreover, the lower values of *n* and ${\eta }$ result in an increment in brightness and vice versa. The relationship between the brightness control parameter ${\eta }$ and intensity is shown in Fig. 3. The curve in Fig. 3 demonstrates that lower values of $\eta$ promote brightness and vice-versa. Where this parameter act as a hyper-parameter to control the brightness directly in the final image without passing through the entire retraining process. The enhancement process follows the congruent decomposition mechanism rather than just inspired by the statistical data. Our clinically oriented enhancement strategy fits well to adjust the irregularities of reflection and inconsistencies of illumination in the target problem. It also disrupts objectionable artifacts, including structure and texture distortions. The intuitive weighting adjustment during training learns to enhance the ill-exposed input images for adjustments in the overall reflection $({S_r})$ and overall illumination components $({S_l})$.

6 $$\begin{aligned} \begin{aligned} S_l{_{(x,y)}}= l{(x,y)}\cdot {w_l} \\ S_r{_{(x,y)}}= r{(x,y)}\cdot {w_r} \end{aligned} \end{aligned}$$

The clinically oriented weighted adjustments in Eq. (5) are tailored in the proposed learnable architecture. It provides a novel paradigm of consistent learning derived through the decomposition of input data. The proposed mechanism’s spatial adjustments and network regularization produce a pleasing visual quality with strong boundaries and edges based on the guidance provided through Eq. (9). The resulting images will significantly contribute to the early diagnosis of many oral dental diseases.

**Figure 5:**
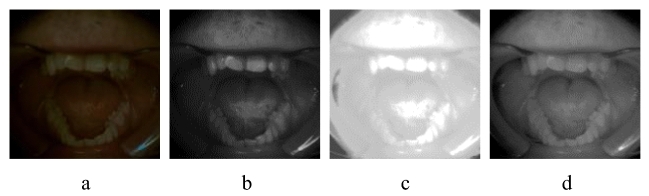
Adjustments in the illumination map for the (**a**) input, (**b**) is Original illumination, and, (**c**) & (**d**) shows the illumination map adaptations.

**Figure 6:**
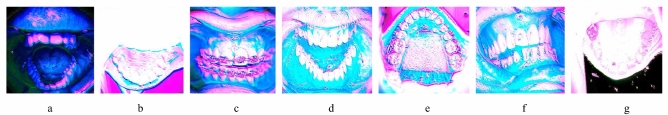
The effects of various color channel-based distortions are shown in (**a**–**g**) for RGB space, handled by the proposed strategy to produce a clean image.

**Figure 7:**
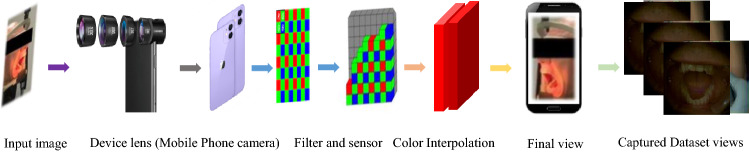
The principle of the proposed image capturing pipeline by using digital capturing devices.

### The proposed denticle edification network

The proposed denticle edification network is end-to-end trained, where it is comprised of two sub-networks, i.e., De-Net and Ed-Net. A multilayered De-Net separates the reflection and illumination components. The splitting process initiates the respective enhancement operations on the input image to update the *r* and *l* components independently. Following decomposition consistency, the proposed clinically oriented enhancement strategy is tailored in the network to mitigate the irregularities of the ill-posed image decomposition. The proposed strategy contributes to limiting objectionable artifacts and preserving the strong features. The input images are decomposed into reflection and illumination components with the help of a denticle net. In De-Net, the feature extraction is initialized with convolutional layers $({3\times 3})$ stack. The features are mapped following the rectified linear unit (ReLU) activation function with another stack of (Conv, ReLU, ${3\times 3}$). In order to provide a distributive adjustment for the decomposed constituents, the hyperbolic tangent function is independently applied across the *r* and *l*.

A tractable image representation is maintained in this network. The decomposed components are upgraded following Eq. (6), and the elementary knowledge based on clinically oriented up-gradation strategy is embedded in the network to update loss functions. The loss functions are designed as distance terms to handle the irregularities of reflection and inconsistencies of illumination. These losses are constrained as the *L*1 norm of reflection and illumination. The overall loss function for the De-Net ${({\mathscr {L}}_{De})}$ is the sum of image split-loss that depicts the decomposition of the subject into *r* and *l* thus denoted as ${({\mathscr {L}}_{slr})}$, the operational loss for reflection regularization $({{\mathscr {L}}_{re}})$ and a clinically adaptive loss (${{\mathscr {L}}_{l_{cal}}}$) for illumination awareness. ${{\mathscr {L}}_{l_{cal}}}$ work under the guidance of the proposed weighting scheme for the target problem, which can promote illumination consistency, thus it is termed as a clinically adaptive loss.

7 $$\begin{aligned} {\mathscr {L}}_{De}={{\mathscr {L}}_{slr}}+\alpha _{re} {\mathscr {L}}_{re}+\alpha _{cal}{\mathscr {L}}_{l_{cal}} \end{aligned}$$

The above equation presents the sum of the loss functions to defy the amplification of the objectionable artifacts, where the values of auxiliary variables ${\alpha _{re}}$ and $\alpha _{cal}$ are 0.004 and 0.3, respectively. The components losses in the above Eq. (7) are adjusted as reflection regularization loss ${l_1}$ norm of the ${\left\| r \right\| _1}$, and image split-loss $({\mathscr {L}}_{slr})$.

8 $$\begin{aligned} {\mathscr {L}}_{slr}={\left\| r_{x,y}l_{x,y}-S_{(x,y)}\right\| }_1 \end{aligned}$$

**Figure 8:**
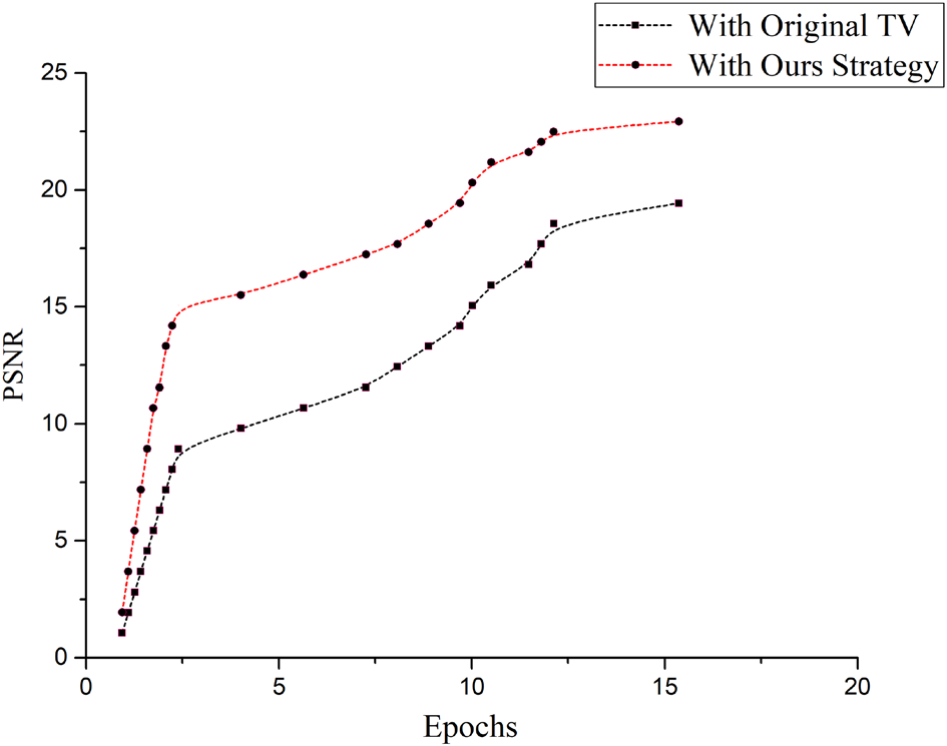
The effects of our strategy on total variation to improve PSNR.

${\mathscr {L}}_{l_{cal}}$ in the above equation is based on the edge-aware illumination consistency achieved in the guidance of the reflection component. The total variation (TV) operation is optimized to serve as a smoothness prior while removing its structure blindness. The corresponding TV loss, as shown in Figs. 4, and 5, disrupt the associated structure and texture artifacts. It can be seen in Fig. 4 that input images in the blue box are enhanced with and without the proposed strategy in the green and red boxes, respectively. In underexposed conditions, the color channel-based distortions can be observed in Fig. 6a to 6g for different input images. To overcome the challenges of illumination inconsistency, an encoder-decoder framework, which is termed an edification network, is proposed. It is important to note that preserving the structure beside strong boundaries and edges is the key constraint for the edification of unevenly exposed input images. It contributes to providing structure and texture preservation with adaptive guidance, where the ${\mathscr {L}}_{l_{cal}}$ in the Eq. (8), is estimated as follows:

9 $$\begin{aligned} {\mathscr {L}}_{l_{cal}}= \left\| \bigtriangledown l_{x,y}\cdot w_l \cdot exp (-\varphi \bigtriangledown (r_{x,y})\cdot w_r) \right\| \end{aligned}$$

Whereas ${\varphi }$=10 provides trade-off control to adjust the gradient. The gradient operator ${\bigtriangledown }$, in the Eq. (9) includes both the horizontal and vertical components expressed as ${{\partial }_h}$, ${{\partial }_v}$ respectively for the *r* and *l*. The smaller derivatives define illumination consistency, and reflection regularities are considered as attributes of the larger derivatives. The impact of the improvements in the image quality due to the proposed TV optimization are shown in Fig. 8, where the impact of the proposed strategy is shown in terms of the PSNR of the network. It clearly demonstrates that the proposed clinically oriented strategy improves the network performance. The key structure and texture adjustments are embedded in the form of loss adjustments in the Ed-net.

The Ed-Net consists of an encoder-decoder unit. The encoder part consists of ${3\times 3}$, upsampling, and the decoder part consists of ${3\times 3}$, downsampling (conv+ReLU), and a stride of 2. Skip-connections maintain spatial consistency, and illumination is upscaled with a ${3\times 3}$ stack of convolutional layers. A multiscale concatenation operation is introduced to maintain the piece-wise illumination smoothness considering the local and global illumination consistency. Moreover, the stack of conv-layers is finally introduced for nearest-neighbor interpolation adjustments across the stride of 1. Our Ed-Net disrupts illumination inconsistencies and induces illumination awareness while obtaining guidance through the clinically adaptive enhancement strategy. The overall loss of this network is adjusted as a sum of ${{\mathscr {L}}_{l_{cal}}}$ and image composition loss ${{\mathscr {L}}_{S_{co}}}$, estimated as below.

10 $$\begin{aligned} {{\mathscr {L}}_{S_{co}}= \left\| {{r_{x,y}}}\cdot {{\widetilde{l}}_{x,y}}-{S_{x,y}}\right\| }_1 \end{aligned}$$

The proposed strategy is clinically adaptive and preserves the image features to induce illumination and reflection consistency. The balanced contrast and enhancement of the luminance in the final image based on the learned adjustments is the distinct feature of the proposed framework. The medical practitioner can have the freedom of luminance adjustments to reveal the hidden features in the images by using the pre-trained model. The adaptive adjustments in the illumination and reflection components finally rectify the hidden artifacts, and at last, the product of reflection and illumination produces an output image. The final image is obtained as a result of enhancement operations tailored to the proposed network. The weighted adjustments in the illumination are obtained as a result of manipulation of various level appointments for the *r* = ${\widetilde{r(x,y)}}$ = $r(x,y) \cdot {w_r}$, and *l* = ${\widetilde{l(x,y)}}$ = $l(x,y) \cdot {w_l}$, without a tedious training process every time. The clinically adaptive weighting strategy provides a user-specific balance of contrast in the final image to visualize the various features in the output for an effective and early diagnosis of many potential dental diseases. Our strategy provides user-specific freedom of choice to adjust the luminance in the final image.

**Figure 9:**
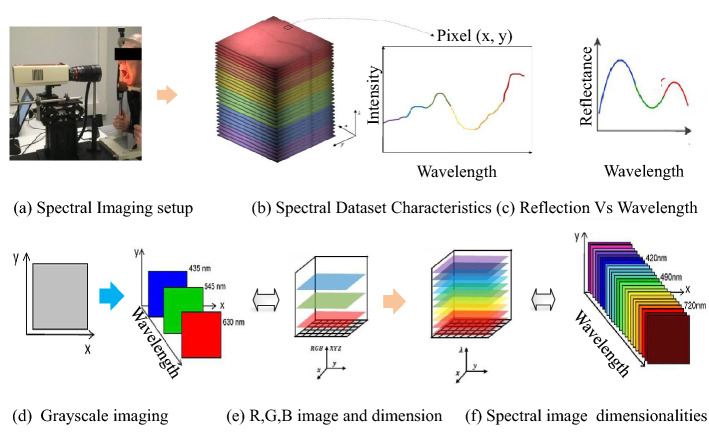
The Spectral imaging setup to characterize the wavelength and reflection distribution, with respect to the color imaging channels and spectral dimensionalities of the data.

**Figure 10:**
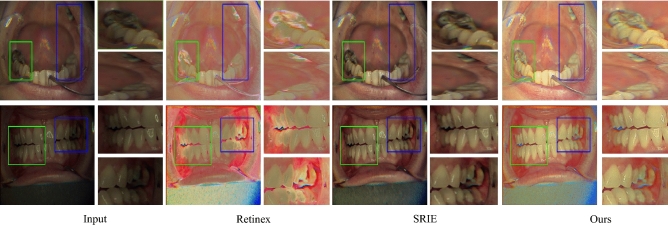
Comparison of the proposed method (ours) with decomposition-based approaches.

**Figure 11:**
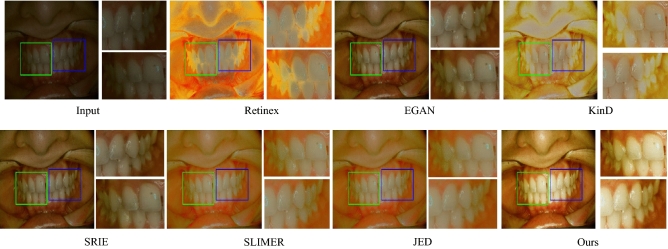
Comparison of the proposed method (ours) with several state-of-the-art approaches.

## Datasets, experiments, and performance evaluation

### Datasets

In the case of dental and oral dental imaging, the healthy and afflicted tissues exhibit various changes in the reflection spectra. Various data acquisition devices are available that store the data files in the grayscale, Red, Green, and Blue (R, G, B) and spectral images, as shown in Figs. 7 and 9. Spectral imaging analysis may provide optimized solutions to detect the changes in dental tissues. The capturing scenario in Fig. 9a–c demonstrates that the weighting adjustments for various bands and wavelengths can optimize contrast with optical filtering^8^. The available spectra can be utilized in training deep learning-based systems^5^, intelligent expert diagnostic systems, and optical imaging systems^8^.

Intelligent learning-based frameworks are becoming popular with the advent of time, but the availability of large-scale datasets in the medical imaging domains is a great challenge. The deep learning-based methods are data-hungry and the rare larger dataset repository in the dental domain. In this work, we utilized the oral and dental spectral image database (ODSI-DB)^8^. The database aims at spectral imaging in oral dental diagnostics. The database contains 316 images of human test subjects. This dataset captured the lower and upper teeth with oral mucosa and face surroundings. To the best of our knowledge, it is the only publicly available database for dental images, and the same has been reported by the authors in^8^.

In this work, we simplify the spectral imaging pipeline as shown in Fig. 9e,f and utilize the ODSI-DB images to train the proposed Ded-Network. In our work, we propose to simplify the capturing assembly, as shown in Fig. 7, which may automate the capturing and diagnostic process. We selected a total of 232 images from the ODSI-DB and converted the Tagged Image File Format (TIFF) to portable network graphics (PNG). Moreover, we separated 16 test images out of these 232 images and utilized these images for subjective and objective evaluation purposes. In this case, a reference ground-truth image is obtained with the help of a dental physician to reveal the true features necessary for the early diagnosis of oral dental diseases. The rest of the images (i.e., 216 images only) are utilized for training the Ded-Net. Training a deep learning-based framework on such a small data sample is challenging. In addition, the adaptation of this framework to various scenarios to detect the desired features for early diagnosis is another challenge. Considering these key challenges, we proposed a framework capable of handling these issues. The subjective and objective evaluations in the next sections demonstrate the superiority of our method.

**Table 1:** Comparison of PSNR and SSIM metrics with several methods.

Methods	LBR	SLIMER	SBLI	BBLLI	JED	Retinex	SRIE	Ded-Net
PSNR ${\uparrow }$	15.6846	17.0082	13.9775	17.6423	18.0482	12.6957	13.97754	22.5831
SSIM ${\uparrow }$	0.8038	0.8628	0.8549	0.8166	0.8148	0.8075	0.85495	0.9368

**Table 2:** The comparison of non-reference image-based quality evaluation metrics.

Methods	LBR	SLIMER	SBLI	BBLLI	JED	Retinex	SRIE	Ded-Net
NIQE ${\downarrow }$	8.7264	7.1658	8.2447	6.5284	7.6905	8.8635	7.0246	5.4237
NQAC ${\uparrow }$	4.0195	4.7683	4.5318	4.3258	4.2718	3.9328	4.5682	5.6792
SIQE ${\uparrow }$	0.6419	0.6742	0.6480	0.6946	0.6836	0.6594	0.6809	0.7186
ASIQE ${\uparrow }$	0.6441	0.6486	0.6518	0.6980	0.6888	0.6605	0.6871	0.7238
LOE ${\downarrow }$	2052	1805	2026	1796	1832	1904	1938	1667

**Figure 12:**
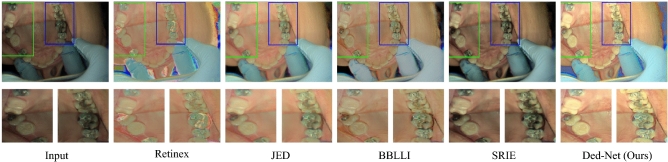
Comparison with Retinex, JED, BBLI, SRIE, and Ded-Net (Ours).

**Figure 13:**
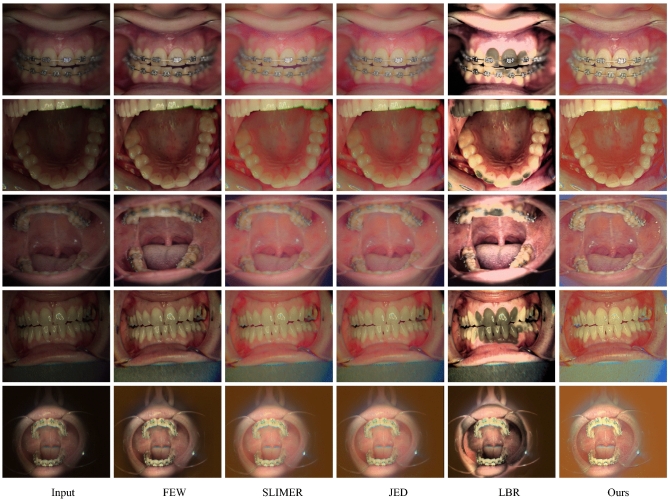
Comparison of our method on input images in the first column with several traditional state-of-the-art approaches.

### Experimental setting

**Network Optimization**: The proposed Ded-net is trained from scratch by using the Tensorflow framework, with Adam optimizer & back-propagation. Ded-Net utilizes a considerably small dataset for training under the guidance proposed scheme to update learning. The training proceeded for 100 epochs, where the patch size is kept ${128\times 128}$ with a batch size of 16 and a learning rate of 0.0001 during the training process. The network gets regularized by utilizing only a few input samples for training only after a few epochs. The network predicts rapidly within a short time and becomes capable of producing adaptive results in various conditions. The overall comparison with the state-of-the-art approaches demonstrates that our method is more practical and suitable in the target domain. The suggested network is simple yet efficient and can be regularized without falling into the local optimal solution. It develops the ability to predict rapidly and precisely under a wide range of conditions.

**Implementation Support**: We utilized a PC core i7,6700K CPU@4GHZ 32GB RAM, NVIDIA 2080Ti GPU to perform the experiments. The comparison with competitor approaches demonstrates that the Ded-Net outperformed the state of the arts in terms of subjective and objective evaluations.

**Figure 14:**
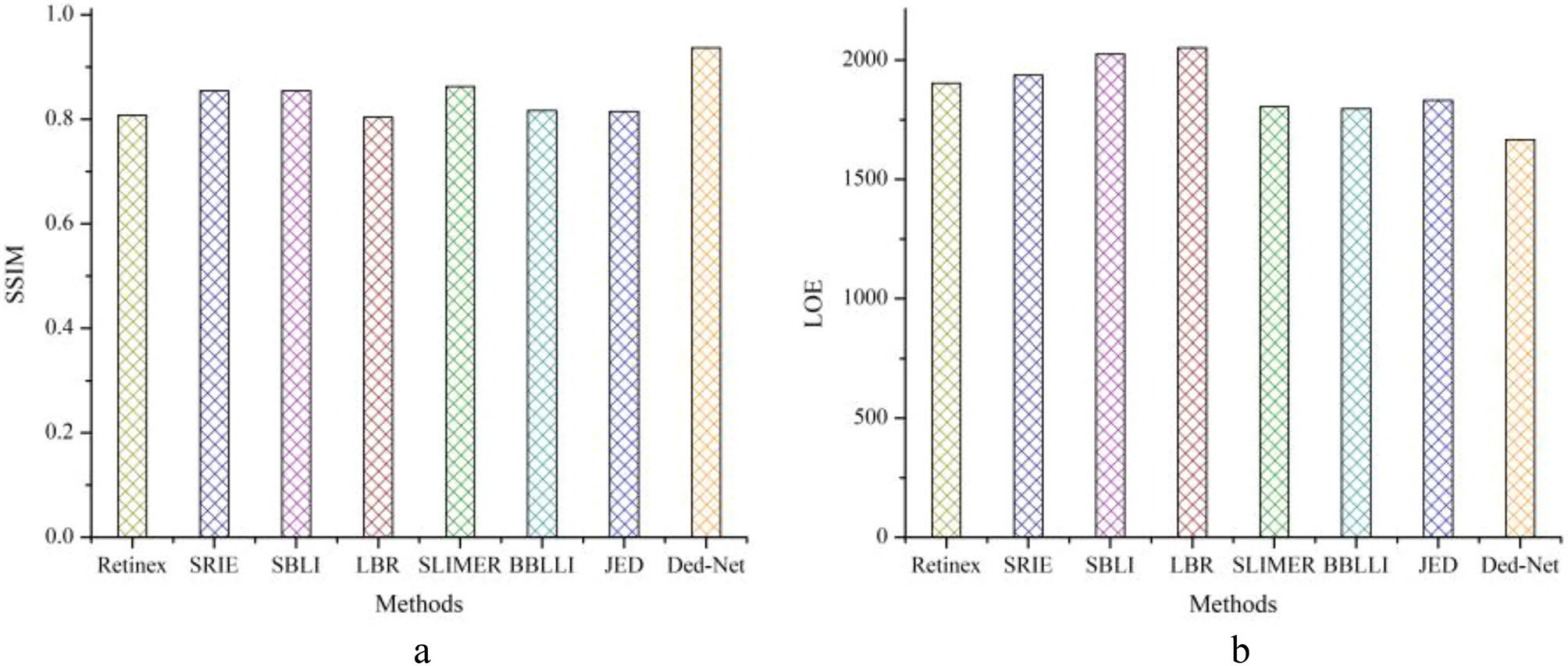
Comparison in terms of (**a**) SSIM and (**b**) LOE evaluation metrics with several sate of the art approaches.

**Figure 15:**
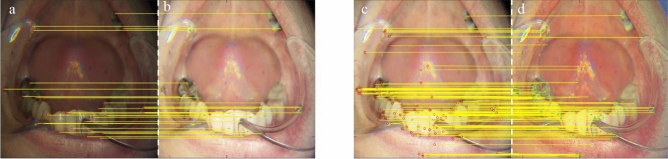
The features matching, (**a**), and (**b**) shows matched feature points for original and ground truth (GT) and, (**c**) and (**d**) shows matched feature points between GT and Ours.

### Experiments

We pre-processed 216 input images and utilized them for training the proposed network. The proposed framework comprehensively resolves the data shortage challenges and is adaptable to robust conditions. The performance of the proposed approach is measured by utilizing the test images separated from the pre-processed images. The challenging images from the available ODSI-DB were selected, and respective ground truth images (GTs) images were obtained. A reasonable visual quality for each image was obtained with manual adjustments in brightness and contrast. Auto-correct operation and brightness adjustment operations were employed to construct reference GTs in the adobe-photoshop. It is why because the full reference image-based objective evaluation metrics (i.e., SSIM and PNSR) require a reference image for the evaluation. In order to generalize the performance, we utilize full reference image-based metrics, besides the non-reference image-based metrics, and also present the visual comparison.

### Subjective and objective performance evaluations

In this section, the proposed method is compared with different state-of-the-art approaches. Subjective results are shown for visual comparison, and objective comparison is based on full reference image-based and non-reference image-based evaluation metrics. To provide an accurate comparison, we utilize structure similarity index measurement (SSIM) and peak signal-to-noise ratio (PSNR) as full reference image-based metrics^48^. A lightness order enhancement (LOE) metric is provided as a non-reference metric for the natural preservation of non-uniformly illumination images^49^.

Similarly, a naturalness image quality evaluator (NIQE), a no-reference quality assessment method for contrast distorted images (NQAC)^50^, a standard and accelerated no reference screen image quality evaluation (SIQE), (ASIQE) methods^51^ are used as non-reference image-based quality evaluation metrics. It is important to note that the reference image-based metrics require a reference ground-truth image (GT), designed in this case with the help of dental physicians, to tackle the exigent glitches. To interpret the quality of the output images, the adaptive enhancement operations are shown in Figs. 2, 4 and 5. The ultimate purpose is to improve the final image quality based on our clinically oriented image enhancement strategy. In this regard, the comparison with the various state-of-the-art approaches is shown in Figs. 10, 11, 12 and 13. The visual comparison in the form of zoomed-in patches demonstrates the superiority of our method.

**Table 3:** Runtime comparison of various methods.

Methods	Runtime	Platform	Methods	Runtime	Platform
SRIE	12	Matlab	LBR	35.5	Matlab
SLIMER	26.5	Matlab	Retinex	0.12	Tf-gpu
JED	7.5	Matlab	KinD	0.002	Tf-gpu
FEW	3.5	Matlab	Ded-Net	0.09	Tf-gpu

The quality of the output of our method demonstrates that our approach provides pleasing visual details. The overall quantitative comparison in terms of SSIM is shown in Fig. 14a and for LOE metric is shown in Fig. 14b. It depicts the overall superiority of the proposed approach in terms of visual and objective evaluations. The proposed improvements in the visual quality are very helpful in the early diagnosis of many oral dental diseases. The proposed adaptive enhancement operations can assist in image-guided robotics surgeries. In our method, we adaptively adjust the color channel-based operation, as shown in Fig. 6, which can assist in the edge preservation and detection tasks. In this Figure, various image samples (a-g) are shown in robust scenarios and suffer different distortions. We produce clean images that significantly improve segmentation and feature detection operations. In Fig. 15, we have shown the improvement in feature detection, but it is out of the scope of this work to explain detection and segmentation, which require a large-scale annotated dataset. To the best of our knowledge, no such dataset is available in the target domain. The proposed Ded-Net mitigates the hidden degradation and produces a pleasing visual balance with an adaptive contrast. The network consistently wipes out the reflection and illumination irregularities as shown in Fig. 2, 4 and 5. The effectiveness of the enhancement operations improves the quality of the output images significantly. The visual comparison and zoomed-in patches demonstrate the effectiveness of the proposed approach.

**Figure 16:**
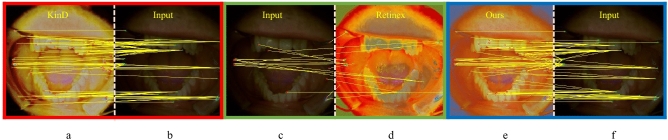
The features matching comparison with Network based methods in red, green and blue boxes. Feature points are matched for (**b**, **c**, **f**) input images. (**a**) shows KinD, (**d**) Retinex and (**e**) shows results for matched feature points for ours.

The images captured with ordinary devices contain low contrast and face several artifacts, including color and contrast distortions. The extant enhancement approaches are hardly suitable for the target problem. Traditional approaches such as SRIE, SLIMER, and LBR methods are suitable for improving the low contrast but have high computational complexity and limited robustness. Such as, the SLIMER produces inconsistent reflection and can hardly handle heavy noises. The JED method proposed in the literature can handle the noises, but it removes the fine details during denoising. Thus the order of denoising plays a key role in enhancing medical imaging to assist the associated operations. Considering these drawbacks, the recent deep learning-based methods are proposed in the literature. However, the deep learning-based methods are data-hungry, and some generative adversarial-based methods, such as EGAN^52^ require a careful designation of the large-scale datasets, whereas other requires image pairs^23^ or very large scale data^53^. In contrast, our framework performs distinctively without any large-scale / carefully designed dataset. Our method is capable of working with minimal latency and memory footprint. The proposed scheme is embedded in the network, where we update the decomposition. It obstructs the amplification of the artifacts and improves the latency. In this way, a correlated consistency is maintained, where the network learns to decompose and update input. The subsequent adjustment operations improve the quality of the final image to extract the necessary details.

Our method consistently produces visually pleasing results with a wide range of adaptability to various practical scenarios. The comparison of our method shown in Figs. 10 and 12 illustrate that the deep retinex method suffers from texture transformation, and SRIE produces inconsistent results. In order to emphasize the zoomed-in patches shown, these patches demonstrate that our method consistently preserves the structure and texture details. The zoomed-in patches in Figs. 10 and 11 show that our method produces the best visual quality as compared to the state-of-the-art approaches. In Fig.10 we compare our method with the decomposition-based methods, and in Fig. 11, we generalize the performance and compare the proposed method with several states of the art approaches. It can be seen in these figures that Some approaches suffer texture transformation, while others result in color distortions besides under-enhancement. Similarly, in Fig. 13, the proposed Ded-Net is compared with several conventional state-of-the-art approaches, i.e., FEW, SLIMER, JED, and LBR methods.

The comparison demonstrates the lack of robustness of the extant methods in the target problem, where our method produces consistent results with more details. The objective comparison on the basis of PSNR, SSIM, and LOE is shown in Table. 1. The comparison based on NIQE, NQAC, SIQE, and ASIQE is shown in Table. 2. The higher values for the PSNR, SSIM, NQAC, SIQE and ASIQE depict higher image quality, whereas the lower values for the LOE and NIQE show higher image quality and vice versa. Moreover, it is important to note that the training time for the several extant deep networks ranges from several minutes to hours. In comparison, the training time for our Ded-Net is less than a minute. The comparison of the computational complexity of the proposed method with several state-of-the-art methods is shown in Table 3. Considering the application-specific requirements, we also present a comparison for feature detection in Figs. 15 and 16. We detect the features based on scale-invariant feature transform^54^. The comparison demonstrates that the proposed method distinctively improves the number of matches for the enhanced image (i.e., Fig. 15c,d) as compared to the original input image (i.e., Fig. 15a,b). In Fig. 16 we compare the proposed method with network based methods, i.e., KinD and retinex. The overall comparison with the state-of-the-art approaches clearly demonstrates that our method is very effective and outperforms the state-of-the-art approaches.

It is also important to note that, to assist the dental physician and robotics operations, minor details can play a key role in diagnosing many oral dental diseases. Thus, considering the target problem’s nature, the proposed clinically oriented enhancement strategy is of immense importance. The results produced by our method are consistent and more adaptive towards various expert system operations, including early diagnosis of several oral dental diseases.

## Conclusion

In this work, a new dental image enhancement framework is proposed for the early detection and diagnosis of oral dental diseases. A practical and clinically oriented adaptive enhancement strategy is proposed to act adaptively in practical scenarios for the early diagnosis of dental diseases. This strategy is embedded in a denticle edification network (Ded-Net) to adjust the degradation of reflection and illumination. A practical trade-off is provided to maintain the balance of luminance in the final image. Unlike previous approaches, the proposed method does not require a large-scale / paired or carefully selected dataset and learns to predict dynamically in resilient situations. The decomposition of the input data into reflection and illumination components facilitates the desired adjustment in the final image, where the network is end-to-end trained to learn from the persistence of decomposition for successive enhancement operations. The proposed framework can also improve the performance of intelligent and expert systems, including many robotic and surgical operations. The overall comparison with the state-of-the-art approaches illustrates the superiority of our method. In future work, we will consider incorporating the proposed framework for some segmentation and detection tasks.

## Data Availability

The dataset used in this work was obtained from the oral and dental spectral image database(https://sites.uef.fi/spectral/odsi-db/).
